# The Number of Catalytic Elements Is Crucial for the Emergence of Metabolic Cores

**DOI:** 10.1371/journal.pone.0007510

**Published:** 2009-10-19

**Authors:** Ildefonso M. De la Fuente, Fernando Vadillo, Martín-Blas Pérez-Pinilla, Antonio Vera-López, Juan Veguillas

**Affiliations:** 1 Department of Mathematics, Faculty of Science and Technology, University of the Basque Country, Vizcaya, Spain; 2 Department of Applied Mathematics and Statistics, Faculty of Science and Technology, University of the Basque Country, Vizcaya, Spain; 3 Department of Physical-Chemistry, Faculty of Science and Technology, University of the Basque Country, Vizcaya, Spain; University of Glasgow, United Kingdom

## Abstract

**Background:**

Different studies show evidence that several unicellular organisms display a cellular metabolic structure characterized by a set of enzymes which are always in an active state (metabolic core), while the rest of the molecular catalytic reactions exhibit *on-off* changing states. This self-organized enzymatic configuration seems to be an intrinsic characteristic of metabolism, common to all living cellular organisms. In a recent analysis performed with dissipative metabolic networks (DMNs) we have shown that this global functional structure emerges in metabolic networks with a relatively high number of catalytic elements, under particular conditions of enzymatic covalent regulatory activity.

**Methodology/Principal Findings:**

Here, to investigate the mechanism behind the emergence of this supramolecular organization of enzymes, we have performed extensive DMNs simulations (around 15,210,000 networks) taking into account the proportion of the allosterically regulated enzymes and covalent enzymes present in the networks, the variation in the number of substrate fluxes and regulatory signals per catalytic element, as well as the random selection of the catalytic elements that receive substrate fluxes from the exterior. The numerical approximations obtained show that the percentages of DMNs with metabolic cores grow with the number of catalytic elements, converging to 100% for all cases.

**Conclusions/Significance:**

The results show evidence that the fundamental factor for the spontaneous emergence of this global self-organized enzymatic structure is the number of catalytic elements in the metabolic networks. Our analysis corroborates and expands on our previous studies illustrating a crucial property of the global structure of the cellular metabolism. These results also offer important insights into the mechanisms which ensure the robustness and stability of living cells.

## Introduction

The cellular metabolism, conformed by the interactions among thousands of enzymes and other biochemical molecules, is a reactive structure densely integrated through an intricate network which constitutes one of the most complex dynamic systems in nature. The molecular structures and the individual topological organization of a great part of the enzymes are well understood, but the characteristics of the functional dynamics that they conform as a whole are still unknown.

In an attempt to get a more accurate comprehension of the global metabolic phenomena, we have developed a reactive dynamical system called dissipative metabolic network (DMN) which is basically formed by groups of enzymatic sets interconnected by several substrate fluxes and allosteric and covalent regulatory signals. Enzymatic allosteric modulation may be both positive (activation of the catalytic rates) and negative (inhibition of the reactive process). The regulation by means of the covalent interactions generates “all or nothing” answers. Each catalytic set of a DMN (called metabolic subsystem or catalytic element of the network) represents a discrete module of several enzymes functionally associated, which may operate within far from equilibrium conditions, and consequently present both steady states and oscillatory reactive patterns (these dynamical behaviors are also called dissipative processes [1]).

Different studies have shown that the enzymes may conform functional catalytic associations [2], [3] e.g., there is evidence that 83% of the proteins of *Saccharomyces cerevisiae* form complexes containing anywhere from two to eighty three proteins [4]; likewise, after assigning individual proteins to protein complexes, these studies have shown the emergence of a higher-order organization structure of the S. cerevisiae proteome conformed by a modular network of biochemical interactions between protein complexes [4].

On the other hand, numerous experimental observations both in prokaryotic and eukaryotic cells have shown the spontaneous emergence of molecular oscillations in the enzymatic processes [5]–[7]. Many of these metabolic oscillations are periodic [5]–[8], for instance, there are oscillatory biochemical processes involved in: biosíntesis of phospholipids [9], cytokinins [10], cyclins [11], transcription of cyclins [12], gene expression [13]–[16], microtubule polymerization [17], membrane receptors activities [18], intracellular amino acid pools [19], membrane potential [20], intracellular ph [21], cyclic AMP concentration [22], respiratory metabolism [23], NAD(P)H concentration [24], glycolysis [25], intracellular calcium concentration [26], metabolism of carbohydrates [27], beta-oxidation of fatty acids [28], metabolism of mRNA [29], proteolysis [30], urea cycle [31], Krebs cycle [32], and protein kinase activities [33].

Likewise, numerous works have been carried out on the mathematical models of metabolic rhythms [1], [6], [8], [and 34].

According to these studies on enzymatic complexes and metabolic oscillations, our DMNs are dynamic systems with interconnected metabolic subsystems which represent enzymatic sets dissipatively structured (their activity may be oscillatory or steady state) and therefore individual enzymatic molecules are not considered in the networks.

In our first work with DMNs we found different global metabolic structures, but particular attention was focused on one of them, characterized by having a set of metabolic subsystems always locked into active states while the rest of catalytic elements present dynamics of *on-off* changing states; it was suggested that this kind of functional self-organization could be common to all living cells [35].

Other studies, implementing a flux balance analysis applied to metabolic networks, produced additional evidence of a global functional structure in which a set of metabolic reactions belonging to different anabolic pathways remain active under all investigated growth conditions, conforming a metabolic core, whereas the rest of reactions belonging to different pathways are only conditionally active [36]–[38]. The existence of the global metabolic structure was verified for *Escherichia coli*, *Helicobacter pylori*, and *Saccharomyces cerevisiae*. The metabolic core is the set of catalytic reactions always active under all environmental conditions, while the rest of the reactions of the cellular metabolism are only conditionally active being turned on in specific metabolic conditions. The core reactions conforms a single cluster of permanently connected metabolic processes where the activity is highly synchronized representing the main integrators of metabolic activity. Two types of reactions are present in the metabolic core: the first type are essential for biomass formation both for optimal and suboptimal growth, while the second type of reactions are required only to assure optimal metabolic performance [37], [38].

In a recent study with DMNs, we observed (under particular conditions of different levels of enzymatic covalent regulatory activity) an asymptotic trend towards 100% of the networks displaying the global configuration with metabolic core when the number of metabolic subsystems is incremented: this suggested that the number of catalytic elements could be the fundamental element for the emergence of the observed global structure [39].

Here, in order to investigate the mechanisms behind the emergence of the self-organized global functional structure we have considered new important elements which were not taken into account in our previous studies. Concretely, we have performed here an exhaustive analysis with around 15,210,000 DMNs, exploring a large rank of topological architectures, taking into account:

- The proportion of the allosteric activation signals, allosteric inhibition signals and regulatory signals of covalent modulation present in the network.
- The variable number of substrate fluxes and regulatory signals that each metabolic subsystem can receive.
- The random selection of the metabolic subsystems that receive substrate fluxes from exterior.

Likewise, we have researched the dynamics of metabolic subsystems functionally unviable, and we have also computed the sizes of metabolic cores, comparing with the results from Almaas et al., [37].

Our analysis reinforces and expands on our previous work and we have concluded that the number of enzymatic complexes (catalytic elements of the networks) is the crucial factor for the emergence of a global functional structure with metabolic cores. Likewise, these studies have allowed us to relate this new structure with the robustness and stability of living cells.

## Results

Dissipative metabolic networks (DMNs) are dynamical systems basically formed by *N* interconnected elements, called metabolic subsystems or catalytic elements of the networks. Each metabolic subsystem represents a group of enzymes aggregated in cluster and dissipatively structured (the catalytic processes can present both stationary and oscillatory activity regimes). These enzymatic sets are considered as individual catalytic entities and receive both input fluxes (the substrates of the enzymatic reactions) and regulatory signals, which may be of three types: activatory (positive allosteric modulation), inhibitory (negative allosteric modulation) and all-or nothing type (which correspond with the regulatory enzymes of covalent modulation).

**Figure 1:**
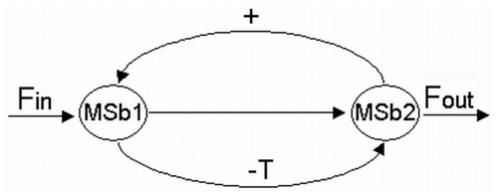
Dissipative metabolic network with only two metabolic subsystems. DMN formed by two subsystems (MSb1 and MSb2) and two feedback loops of regulatory signals. Each metabolic subsystem represents a discrete module of several functionally associated enzymes, which may operate in far from equilibrium conditions, and consequently may present both steady states and oscillatory catalytic patterns. The MSb1 receives an outer input flux of substrate and is activated by the second subsystem (+) in which there is an allosteric enzyme of positive modulation. In the MSb1 there is a regulatory enzyme of covalent modulation and the MSb2 may be totally inhibited by the first subsystem (-T).

As an example, we have first considered a simple DMN formed by two subsystems (MSb1 and MSb2) arranged in series with two feedback loops of regulatory signals (figure 1). The metabolic subsystem MSb1 receives an outer input flux of substrate and is activated by the second subsystem (in the MSb2 there is an allosteric enzyme of positive modulation). The MSb2 is totally inhibited by the first subsystem (in the MSb1 there is a regulatory enzyme of covalent modulation).

We have fixed as control parameter the δ threshold value in the regulatory signals of total inhibition which represents the level of the enzymatic covalent regulatory activity. The rest of the network parameters are described in the example of the materials and methods section.

After the numerical resolution, at small threshold values, for 0≤δ≤0.37, the MSb1 presents a single oscillatory behaviour of one-period and the second metabolic subsystem is inactive (the small threshold values represent a high level of the enzymatic covalent regulatory activity which provokes the total inhibition of the MSb2).

A qualitative change in the dynamical structure of the network emerges for 0.38<δ<0.70: the MSb1 is always locked in an *on* state while the MSb2 is in an *on-off* switching state.

The dynamical patterns that emerge for these δ values are simple. For 0.38≤δ≤0.51 the first subsystem exhibits transitions between two periodic oscillations and the MSb2 presents one steady state when it is active. This steady state is replaced for one periodic pattern in the range of 0.51<δ≤0.61.

**Figure 2:**
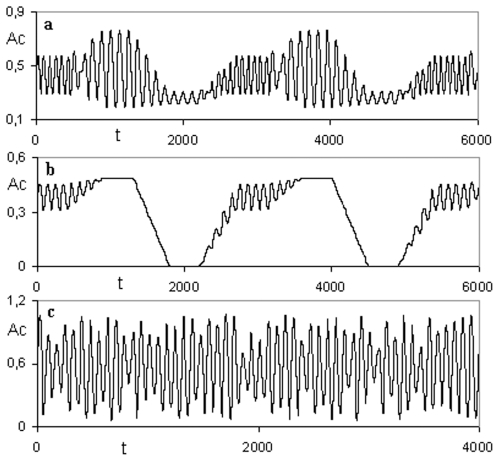
Dynamical catalytic patterns in a DMN formed by two subsystems. (a) In the MSb1 a cycle of three periodic oscillations emerges. (b) The MSb2 presents an *on-off* switching state under the same parametric conditions as the MSb1 and exhibits regular transitions between a periodic pattern and a steady state when it is active. (c) In the MSb1 deterministic chaotic patterns can be observed; the metabolic subsystem modify uninterruptedly their catalytic activity so that it never repeats itself for arbitrarily long periods of time. The activity Ac developed by each metabolic subsystem is represented as a function of the time t.

A third periodic behaviour appears in the range 0.61<δ≤0.68 in both metabolic subsystems. The MSb1 exhibits three periodic oscillations and the second subsystem presents transitions between a periodic pattern and a steady state when it is active (figure 2).

The modification of the control parameter in the range of 0.68<δ≤0.70 leads to the emergence of a new periodic oscillation in both subsystems (four oscillations in the MSb1 and 2 periodic patterns and one steady state in the MSb2).

When the parameter achieves a determinate value, 0.70<δ≤1, the two subsystems are always active. For 0.70<δ≤0.75 the MSb1 exhibits four periodic oscillations and the Msb2 presents transitions between a steady state and a periodic pattern.

As δ increases the temporal structure of the metabolic subsystems becomes more complex with cycles of transitions with 8, 12, 30, 46 etc., patterns.

Finally, for 0.9<δ≤1 deterministic chaotic transitions can be observed (figure 2). The network formed by only two metabolic subsystems spontaneously auto-organizes, provoking the emergence of a very complex behavior in which each subsystem presents infinite transitions between different periodic patterns. In this dynamical situation, both metabolic subsystems modify uninterruptedly their activity so that it never repeats itself for arbitrarily long periods of time.

The mechanism that determines these behaviours in both subsystems is not prefixed in any of the parts of the system. There is neither feedback with oscillatory properties nor other rules that determine the system to present complex transitions in the output activities of the metabolic subsystems. The complex dynamic behaviours which spontaneously emerge in the network have their origin in the regulatory structure of the feedback loops, and in the nonlinearity of the constitutive equations of the system.

In this paper, our main goal is to investigate the mechanism behind the emergence of the supramolecular organization of the cellular metabolic activities.

There are two fundamental kinds of processes that may affect the global functional structure which should be taken into account in our study: the internal metabolic activities of the cells during the growth conditions and the different metabolic structures that characterize the distinct cell types.

The studies of Almaas et al., [37], [38] have shown that the internal metabolic activities are adjusted to environmental changes through two distinct mechanisms:

Flux plasticity, involving changes in the active catalytic reactions when the organism is shifted from one growth condition to another; these reactive reorganizations result in changes in the intensity of the substrate fluxes.

Structural plasticity, resulting in changes in the dynamic structure of substrate fluxes that present *on-off* changing states, so previously inactive catalytic processes are turned on while previously active pathways are turning off and viceversa.

We have considered these processes by means of random changes in: the parameters associated to the flux integration functions, the coefficients of the regulatory signals, and the initial conditions in the activities of all metabolic subsystems. Likewise, we have taken into account the external perturbations by means of random changes in the values of the outer flux parameters and by performing a random selection of the metabolic subsystems that receive substrate fluxes from the exterior.

The different metabolic structures that the distinct cellular types may present have been considered by means of changes in: the proportion of the allosteric activation signals, allosteric inhibition signals and regulatory signals of covalent modulation, the variable number of substrate fluxes and regulatory signals that each metabolic subsystem can receive, the number of metabolic subsystems, the number of input fluxes for each subsystem, the number of input regulatory signals for each metabolic subsystem and the topology of all flux and regulatory signal interconnections.

Taking into account these metabolic elements, we have performed here an exhaustive analysis with around 15,210,000 DMNs in order to simulate extensively different metabolic conditions and to investigate the mechanisms behind the emergence of the self-organized global metabolic structure.

**Figure 3:**
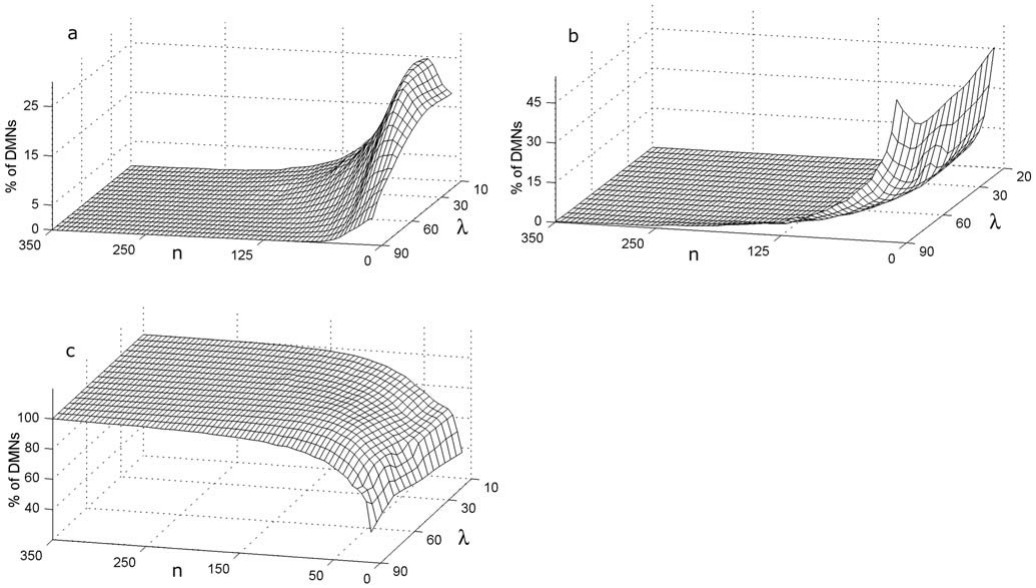
Effect of the number of subsystems on the three main global functional structures. Percentage of DMNs that exhibit, respectively, (a) all their catalytic elements in an *on-off* regime, (b) all the metabolic subsystems unable to change the state and (c) a set of metabolic subsystems conforming a metabolic core while the rest of catalytic elements exhibit an *on-off* changeable state. In the horizontal axes the λ value, the proportion of allosteric and covalent regulatory signals and the number of subsystems *n* are displayed (a 10% of λ means that a 10% of the overall regulatory feedbacks in the network are allosterics signals and the 90% remaining are regulatory signals of covalent modulation). In these analyses, all the DMNs were performed with three regulatory signals and two input fluxes per metabolic subsystem.

We first studied 2,700,000 random metabolic networks whose only common characteristic is having three regulatory signals (*rs* = 3) and two input fluxes (*f* = 2) per subsystem (figure 3), with the goal of measuring what percentage of the networks exhibit global self-organization depending on the proportion of allosteric and covalent regulatory signals per metabolic subsystem λ, and the number of subsystems *n*.

The first control parameter λ was varied in steps of 10 in the interval [10%, 90%] (i.e., a 10% of λ means that 10% of the overall regulatory feedbacks in the network are allosterics signals and the remaining 90% are regulatory signals of covalent modulation).

To simplify, we have considered the allosteric signals (activators or inhibitors) in equal proportion, i.e., if λ = 30% means that a 30% of the overall regulatory feedbacks in the network are allosterics signals provoking that in a 15% of the metabolic subsystem activities can be enhanced, in other 15% can be inhibited.

In the DMNs, three fundamental kinds of global configurations may emerge (see [39] for more details): all metabolic subsystems always present cycles of activity-inactivity (the corresponding analysis about their emergence are in figure 3a), all catalytic elements are unable to change their state (each subsystem is always *on* or is always *off*) (figure 3b), and networks characterized by the presence of a metabolic core (figure 3c).

The first analysis shows how the DMNs that exhibit the two first global configurations (figures 3a and 3b) display a fast decrease in the percentages of networks without metabolic cores as a function of the number of subsystems, converging to 0% for all λ values. In particular, in the networks with all subsystems always *on-off* (figure 3a) and a high number of allosteric signals (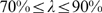) the approximations that we have calculated are equal to 0% when *n* = 180 and the rest of networks with less percentage of allosteric signals per subsystems (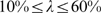) makes it for 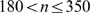; the DMNs with all catalytic elements unable to change their state (figure 3b) becomes 0% for 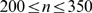 except when λ = 90% which decreases their percentage very slowly in functions of *n* being the value obtained in our approximations of 0% for *n* = 700 (this point is not represented in the figure 3b). However, the analysis shows a fast growth in the percentage of networks with metabolic cores (figure 3c) as a function of the number of subsystems, converging slowly to 100%. So, for *n* = 130 the networks go over the 95% threshold, and all the DMNs present these kind of global structure when the nets reach *n* = 350 elements, except for λ = 90%, in which case the percentage varies very slowly as a function of *n*, reaching 99.5% for n = 350 and 100% for *n* = 700.

In each estimate of the percentages of DMNs the average of 10,000 random metabolic networks were calculated, and the criterion followed to determinate the activity of each metabolic subsystem was to take the corresponding state between the iterations 200 and 300. All the percentages have been estimated according to these averages with groups of 10.000 different DMNs; therefore our results are an approximation to the exact value.

**Figure 4:**
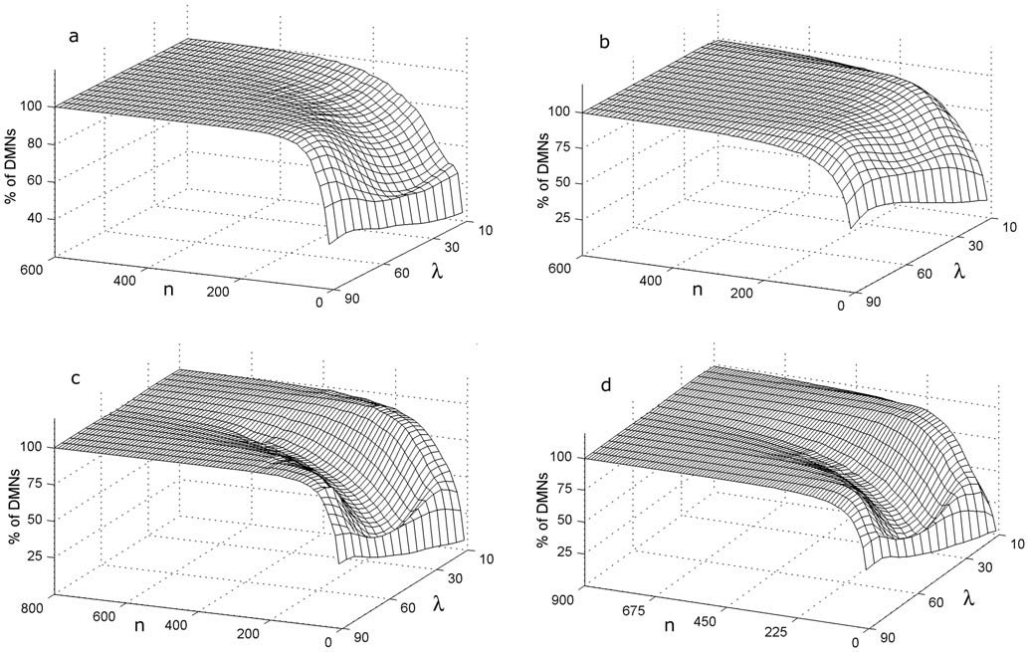
Analyses of DMNs with different number of regulatory signals and fluxes per metabolic subsystem. Percentage of DMNs that exhibit metabolic cores belonging to four kind of metabolic networks with different number of regulatory signals (*rs*) and the fluxes (*f*) per catalytic element: *rs* = 4 and *f* = 2 (figure a), *rs* = 5 and *f* = 3 (figure b), *rs* = 7, *f* = 3 (figure c) and *rs* = 8, *f* = 4 (figure d). In the horizontal axes the λ value, the proportion of allosteric and covalent regulatory signals per network and the number of metabolic subsystems *n* are represented.

Next, to analyze the emergence of these global structures in more complex situations, similar statistical measurements were also carried out with four groups of DMNs in which different relationship between the number of regulatory signals (*rs*) and the fluxes (*f*) per subsystem were taken into account (i.e., *rs* = 4 *f* = 2, *rs* = 5 *f* = 3, *rs* = 7 *f* = 3 and *rs* = 8 *f* = 4, figures 4a to 4d).

In Figure 4a the random DMNs (belonging to the kind *rs* = 4 and *f* = 2) show an increase in the percentage of networks with metabolic cores as a function of the number of subsystems until to converge slowly to 100%. The networks with others global functional structures without metabolic cores goes disappearing as the number of subsystems increases. The percentages for *n* = 5 are 3.5% (λ = 10%) and 21.4% (λ = 90%), and for *n* = 290 all the nets to overcome the 95%.

In the Figure 4b it can be seen that the percentages of DMNs (of the kind *rs* = 5 and *f* = 3) with metabolic cores grow with the number of subsystems for all λ values, and for *n* = 600 our approximations allow to observe that the networks, independently of their structural topology of fluxes and regulatory signals, acquire an only global structure with metabolic cores. The percentages for n = 10 are 12% (λ = 10%) and 44.4% (λ = 90%). The rank of growth for the first 100 metabolic subsystem (that is, the difference of the percentage between *n* = 100 and *n* = 10) are 59.6 for λ = 10%, and 45.4 for λ = 90%. For n = 400 all the DMNs with metabolic cores to overcome the 95%.

In figure 4c, like previous analyses, the percentages of DMNs with cores simulated with seven regulatory signals and three input fluxes per subsystem (rs = 7 f = 3) converge slowly to 100% at n = 800. The minimum percentages are 6.4% (n = 10 and λ = 10%) and 43.9%. (n = 10 and λ = 90%). For n = 550 all the nets surpass the 95% level.

Finally, the figure 4d also allows to observe that the DMNs (of the kind *rs* = 8 *f* = 4) conformed by high *n* values independently of their structural topology of fluxes and regulatory signals acquire an only global dynamic configuration characterized by present a metabolic core. The minimum percentages are 2.7% (*n* = 10 and λ = 10%) and 45.8%. (*n* = 10 and λ = 90%). The rank of growth for the first 100 metabolic subsystem (the difference of the percentage between *n* = 100 and *n* = 10) are 40.5 for λ = 10% and 45.8 for λ = 90%. As it happened in the previous analyses, not all the networks require the same number of catalytic elements to converge to 100%, some of them require a smaller number of metabolic subsystems, for example some percentage of the networks with cores to overcome the 95% for *n* = 220, λ = 20% and 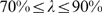.

In our analyses, we have observed large deviations in the percentages of networks with metabolic cores as a function of the number of subsystems for intermediate ranges of λ, e.g., in figure 4 for n = 200 and λ = 50% the values are the following ones: (4a) *rs* = 4 *f* = 2, 94.5%; (4b) *rs* = 5 *f* = 3, 99%; (4c) *rs* = 7 *f* = 3, 70.6%; and (4d) *rs* = 8 *f* = 4, 69%.

The analyses with DMNs also show that the structural levels of allosteric and covalent regulatory activity affect to the size of the metabolic cores and consequently to the percentage of catalytic elements locked into an *on-off* switching state (Table 1). It should be stressed that both the subsystems always *on* and always *on-off* are parts of the same whole, dynamic reactive organization and that one kind cannot exist without the other.

**Table 1:** Size of the metabolic cores.

λ	*f* = 2 *rs* = 3	*f* = 2 *rs* = 4	*f* = 3 *rs* = 5	*f* = 3 *rs* = 7	*f* = 4 *rs* = 8
10%	6.3%	2.2%	11.3%	11.4%	16.8%
20%	6.%	1.7%	7.6%	4.7%	6.7%
30%	5.9%	1.5%	5.6%	1.9%	2.5%
40%	6.4%	1.6%	4.6%	1%	1.1%
50%	7.6%	1.9%	4.6%	0.7%	0.7%
60%	9.8%	2.5%	5.7%	0.9%	0.9%
70%	13.8%	3.8%	8.9%	1.6%	2%
80%	21.5%	6.3%	17.6%	4.%	6.9%
90%	36.8%	13.2%	44.6%	19%	37%

Percentages of catalytic elements per network always active. The λ value is the proportion of allosteric and covalent regulatory signals; *rs* and *f* represent the number of regulatory signals and fluxes per catalytic element. The data are the average percentage of 10,000 random metabolic networks for a fixed λ and for *n* = 500.

The sizes of the metabolic cores (percentages of catalytic elements that are always active) ranged between 44.6% and 0.7% and their distribution is skewed toward low values since 71% of the studied networks show sizes that range between 7.6% and 0.7%, with a mean of 2%.

The lowest values of the sizes correspond to the DMNs with bigger number of regulatory signals and fluxes per catalytic element and similar proportion of allosteric and covalent regulatory signals per metabolic subsystem (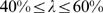). The maximum values of the core sizes emerge when in the overall of the networks most of the regulatory signals are of covalent modulation (λ = 90%).

As expected, we have found that the DMNs studied inevitably present catalytic elements always in an inactive state because certain random connections in the fluxes and regulatory signals convert them in functionally unviable.

In fact, the percentage of these inactive subsystems may be notably large in the nets that contain few subsystems (i.e., for *rs*:7-*f*:3, *n* = 10 and λ = 10% is a 71.29% and for *rs*:5-*f*:3, *n* = 10 and *λ* = 10% is a 68.4%). However, the numerical results show how, when the number of subsystems increases, the percentage of catalytic elements always inactive descends quickly, trending asymptotically to zero.

**Figure 5:**
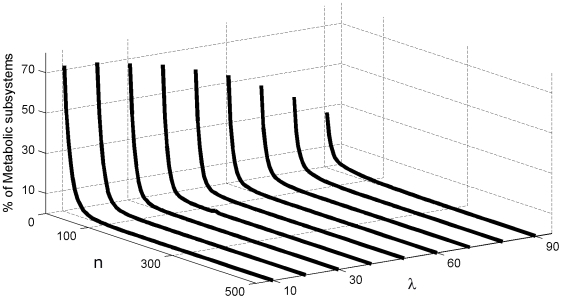
Effect of the number of metabolic subsystems on the catalytic elements always inactive. The figure displays the percentages of the metabolic subsystems always inactive per network as a function of the proportion of allosteric and covalent regulatory signals λ, and the number of subsystems *n*. When the number of the catalytic elements *n* increases the percentage of metabolic subsystems always inactive descends quickly, trending asymptotically to zero. These numerical analyses correspond to DMNs with four regulatory signals and two input fluxes per catalytic element.

The figure 5 shows an example of this dynamical behaviour. The maximum percentage of the catalytic elements always inactive is 72.6% for λ = 10% and *n* = 5 descending to 0.003% for *n* = 380. The DMNs with lower values of λ (consequently with bigger percentage of covalent modulation regulatory signals) and small *n*, present higher percentages of subsystems always *off*.

In all analyses, for large *n* values, the numerical evidence shows that, on the one hand, the percentage of catalytic elements always inactive descends quickly, trending asymptotically to zero and, on the other hand, virtually all random networks spontaneously acquire a unique global dynamic configuration characterized by presenting a metabolic core, and that the approximations that we have obtained are 100% for all λ values. These data seem to indicate that the crucial element for the emergence of a global functional configuration characterized by presenting a metabolic core is a high number of metabolic subsystems.

## Discussion

To investigate the mechanism behind the emergence of a supramolecular organization of the cellular metabolism characterized by the emergence of a sets of enzymes which are always in an active state (metabolic core), while the rest of the molecular catalytic reactions exhibit *on-off* changing states we have analyzed extensive DMN simulations, exploring a wide variety of topological architectures with different allosteric and covalent regulatory conditions under different numbers of substrate fluxes and regulatory signals per each metabolic subsystem.

Our results show that the percentages of DMNs with metabolic cores grow with the number of subsystems converging to 100% for all cases. These results seem to indicate that this global structure is an emergent property which arises in all the dissipative metabolic networks with a relatively high number of metabolic subsystems, independently of their structural topology of fluxes, regulatory signals and the different number of allosteric and covalent enzymes present in each network.

These data corroborate and expand on our previous studies [39]. Therefore it can be concluded that the fundamental element for the spontaneous emergence of a global metabolic configuration, characterized by presenting a metabolic core, is the number of metabolic subsystems belonging to the network.

In cellular conditions, the enzymes and the rest of proteins present multiplicity of copies. For instance, the metabolic network of E. coli K12, one of the best studied cellular organisms, only presents 4,377 genes, 4,290 encode proteins of which 931 correspond to enzymes [40] organized in the cytoplasm and membranes in 165 metabolic pathways [41]. Despite this small number of genes, the number of all synthesized proteins including the enzymes is very high about 3,600,000 (1,000,000 of them being of cytoplasmic proteins, excluding 900,000 ribosomal proteins which under different growing conditions conform between 10,000 and 60,000 ribosomes) [42]. Likewise, the inner membrane proteins are about 200,000 and the outer membrane proteins are about 300,000 [42].

On the other hand, Gavin et. al., in [4] found that an 83% of proteins of *Saccharomyces cerevisiae* form complexes containing anywhere from two to eighty three proteins; over 90% of these aggregates had at least one component that was not found in other sets and the whole enzymatic structure is conformed by a modular network of biochemical interactions between protein complexes.

Our numerical results show that the emergence of the global structure with a metabolic core depends on the number of dissipatively structured enzymatic sets.

The high multiplicity of copies of the enzymes and the corresponding multiplicity of metabolic subsystems could ensure the spontaneous emergence of the cellular metabolic structure.

We have found that the sizes of the metabolic cores (percentages of catalytic elements that are always active) ranged between 44.6% and 0.7% (table 1) and their distribution is skewed toward low values since 71% of the studied networks show sizes that range between 7.6% and 0.7%, with a mean of 2.%.

By means of flux-balance analysis the percentage of enzymatic reactions belonging to the core was 36.2% of all different reactions in H. pylori (138 of 381), 11.9% in E. coli (90 of 758) and 2.8% in S. cerevisiae (33 of 1,172) [36], [37].

In our numerical simulations we have taken into account tree kinds of fundamental changes in the DMNs that may affect the emergency of the observed global metabolic structure: the internal activity of the metabolic subsystems, the external perturbations and the structural changes that can affect the networks.

We have looked at more than 15 million different networks, and the changes in the internal activity of the metabolic subsystems, the structural changes in the DMNs and the external perturbations do not make the functional structure disappear.

Our results indicate that despite the presence of these perturbations, a relatively high number of catalytic elements ensure the emergence of the global metabolic self-organization characterized by a metabolic core in all the cases that we have analyzed. When the networks achieve a determinate number of subsystems they maintain theirs global funcional configurations despite external and internal perturbations.

The DMNs conformed by a relatively high number of subsystems seems to be robust dynamical systems because their topological organization and their network constituent elements can be altered strongly with very little consequence for the overall functional structure.

Robustness is particularly advantageous to cellular organisms. On one hand, it is fundamental to allow the maintenance of the global functional structure despite of the environmental perturbations. On the other hand, this property is considered to be fundamental to facilitate evolvability [43].

The stability of a global reactive structure with metabolic core contrasts with the instability of chaotic patterns which can emerge in certain metabolic processes at the cellular level (e.g., in intracellular free amino acid pools [19], respiratory metabolism [44], photosynthetic reactions [45], glycolysis [46], krebs cycle [47], peroxidase-oxidase reactions [48], membrane potential [49], nuclear translocation of the transcription factor [50], NAD(P)H concentration [51], cyclic AMP concentration [52], ATP concentration [53], intracellular calcium concentration [54]).

DMNs also exhibit simultaneously stable global configurations with metabolic cores and different steady states, regular oscillations and chaotic reactive transitions in the activity of different metabolic subsystems [39].

The existence of chaotic dynamics in the activity of some metabolic subsystems integrated in a robust global functional structure may constitute a biological advantage since the sensitivity of the reactive chaotic behaviors to initial conditions may permit fast and specific metabolic responses during adaptation to the environmental perturbations.

The high number of catalytic elements that characterize the living cells seems to be the fundamental element for the emergence of a singular *cellular metabolic structure* able to self-organize spontaneously, conforming a metabolic core of reactive processes that remain active under different growth conditions while the rest the molecular catalytic reactions exhibit structural plasticity.

Understanding the elemental principles governing the cellular metabolic structure as well as their nexus with central cytological processes may be one of the most important goals of the post-genomic era.

## Methods

### 1. Dissipative Metabolic Networks Model

The model takes into account the fact that the cellular organization at the molecular level presents two relevant dynamic characteristics: the presence of enzymes aggregated in clusters and the emergence of dissipative catalytic patterns.

In agreement with these considerations, the dissipative metabolic networks are dynamical systems basically formed by a given number of interconnected elements, called metabolic subsystems or catalytic elements of the network, each of which represents a modular set of enzymes aggregated in clusters whose activity can present steady state patterns or nonlinear periodic oscillations with different levels of complexity comprising an infinite number of distinct activity regimes.

We assume that the activity of the i-th metabolic subsystem is defined by
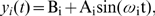
where *A_i_* is the amplitude of oscillation, 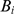 is the baseline and 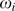 is the oscillation frequency. Moreover, in order that 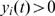 we assume that 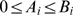 and we also suppose that the means and the frequencies are bounded values, so there exist 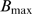 and 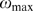 such that
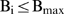
and
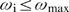


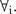
In this way, the activity of each metabolic subsystem 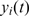 can be characterized by three variables 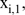 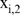 and 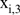, with values between 0 and 1 such that
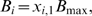


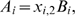


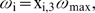
The subsystem is inactive when 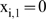, and is in a steady state when 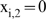 or 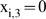.

Fix 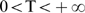 and let 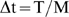 be a bit time interval during which the oscillations are constant, in the m-th time interval between 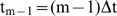 and 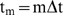 the activity of the i-th subsystem is represented by the vector 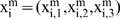 and the state matrix
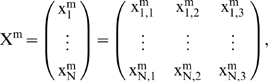
characterizes the whole DMN system, where *N* is the total number of subsystems.

To study the evolution of the whole system, we assume that each subsystem receives two different kinds of inputs:

- The substrates of the biochemical reactions.
- Regulatory signals of three types: activatory, inhibitory and total inhibitory.

These inputs may produce a change in the activity and regulatory signals sent to other subsystems. Moreover, according to experimental observations from [33], the output activity must be stationary or periodic.

Each subsystem processes inputs to produce outputs in two stages:

1. An intermediate activity is obtained using the flux integration functions.
2. The received regulatory signals originate a regulatory signal integration which varies the intermediary activity.

### 2. Flux integration

Let us suppose that the i-th subsystem receives flux from the j-th, its intermediate values 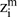 will be computed by three flux integration functions
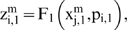


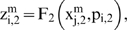


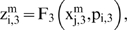
Where 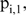 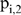 and 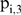 are parameters associated to the flux integration function which are characteristic of each metabolic subsystem, and the *Fi* are piecewise linear approximations for nonlinear functions obtained in [55] by Goldbeter and Lefever in their studies about the oscillations for glycolytic subsystems. In this paper, the functions will be the following:
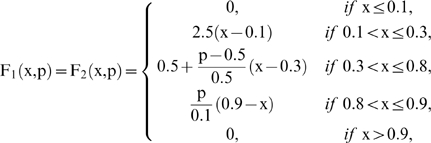
and
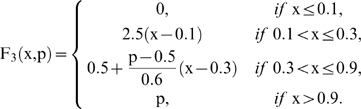
When a subsystem receives flux from at least two subsystems, we compute the arithmetic mean.

### 3. Regulatory signal integration

In this second stage, the intermediary values are modified using the signals integration functions, which depend on the combination of the received regulatory signals and their corresponding parameters (regulatory coefficients). In the metabolic subsystems, the existence of some regulatory enzymes (both allosteric and covalent modulation) permits the interconnection among them. The allosteric enzymes present different sensitivities to the effectors, which can generate diverse changes on the kinetic parameters and in their molecular structure; likewise, the enzymatic activity of covalent modulation also presents different levels of regulation depending on the sensitivity to other activators or inhibitors.

These effects on the catalytic activities are represented in the dynamical system by the regulatory coefficients and consequently each signal has an associated coefficient which defines the intensity of its influence. There exist three kinds of signals integration functions:

- Activation function AC.
- Inhibition function IN.
- Total inhibition function TI.

In this way, to compute 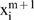 from 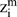 the i-th subsystem receives enzymatic regulatory signals from r subsystems and they work sequentially computing
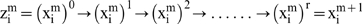
where each step depends on the signal type. From 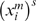 to 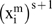 if the signal is AC and is received from the j-th MSb
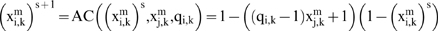
for *k* = 1; 2; 3 and 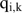 are regulatory coefficient to each allosteric activity signal which represents the sensitivity to the allosteric effectors.

If the allosteric signal is inhibitory
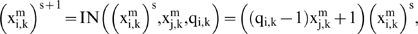
and, finally, if the signal is of the total inhibition type
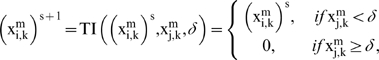
where δ, the threshold value, is the regulatory coefficient associated to each enzymatic activity signal of covalent modulation which defines the intensity of its influence.

### 5. Random metabolic network generations

First, we have fixed with control parameters the following elements:

- The number of subsystems in the DMN.
- The number of input fluxes for each subsystem (in this paper the number of output fluxes for each subsystem is not a fixed parameter so that the metabolic subsystems can present different outputs, (as it happens in cellular conditions).
- The number of input regulatory signals for each metabolic subsystem (similar considerations as with output fluxes have been applied for regulatory output connections). These regulatory signals can come from any element of the network and do not require any flux relationship. Therefore, there are not elements with single fluxes and all the metabolic subsystems have fluxes and regulatory signals.
- The proportion of the allosteric activation signals, allosteric inhibition signals and regulatory signals of covalent modulation present in the network (λ parameter).
- The proportion of the metabolic subsystems receiving substrate fluxes from the exterior (one external flux per metabolic subsystem).

Having fixed these elements, the structure of each network has been randomly configured (following the uniform distribution) including: the topology of flux interconnections and regulatory signals, the 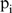 parameters associated to the flux integration functions, the 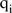 regulatory coefficients to the allosteric activities, the selection of the metabolic subsystems that receive substrate fluxes from the exterior (here 8% of the total), the values of the outer flux parameters 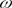, *A* and *B* as well as the initial conditions in the activities of all metabolic subsystems.

The values of 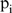 and 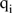 are a random number between 0 and 1. The changes in the parameters 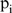 modify the flux integration function, which are piecewise linear approximations for the nonlinear functions obtained in [56] by Goldbeter and Lefever. The values of 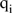 close to 0 represent a low level of influence of the allosteric regulatory signals, and the values of 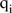 close to 1 represent a high level of influence of the allosteric regulatory signals. The random value of these 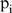 and 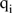 parameters originates metabolic networks with a great variety of activities in each subsystem.

We have taken the constants 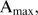 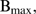 and 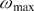 equal 2, anyway, in this paper only the qualitative aspects of the activity have been considered (here, we are mainly interested in the percentages of DMNs with or without metabolic cores, i.e.: if the subsystem is active or inactive, and this is independent of 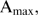 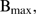 and 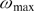 values).

Finally, given *T* and *M* we calculate the activity matrices 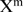 for *m* = 1, …, *M* using the flux integration functions and the regulatory signals.

### 6. Activity of the metabolic subsystems

We consider a certain number of transitions and, in the *k-th* stage, the activity of the metabolic subsystem is described by a function of the form 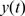 = 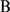
+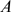
sin(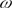
t), where 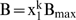, 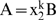 and 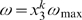 and where 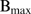 and 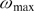 are given fixed parameters independent of the stage number and of the subsystem. The duration of the harmonic oscillation is a given parameter 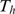 independent also of the stage and of the subsystem. In between two stages, a mixed transition regime is maintained with a duration 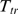 independent of the stage number and of the subsystem. If the transition goes from the *k-th* stage to the *(k+1)-th* stage then, during the 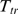 seconds of transition regime, the activity is given by a function of the form 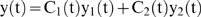, where 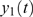 is the activity corresponding to the prolongation in time of the previous harmonic activity in the *k-th* stage, and 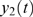 is the back-propagation in time of the subsequent harmonic activity in the *(k+1)-th* stage. The numbers 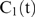 and 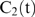 depend on time and indicate the weights with which the activities of the subsystem in the previous and posterior stage are present during the transition time. At the beginning of the transition, say at 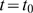, 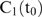 is 1 and 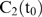 is 0, and at the end of the transition, say at 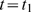, 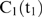 is 0 and 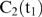 is 1. In the rest of the transition times 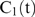 and 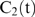 vary affinely. Thus, 
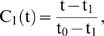
 
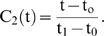
 Putting all this together, during the transition time the activity is given by
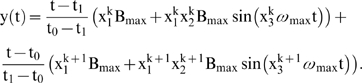
The transition regimes are combinations of two harmonic oscillations with non-constant coefficients 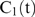 and 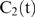 depending on time. Thus, the introduction of these transition regimes provokes the emergence of nonlinear oscillatory behaviors.

### 7. Example

We will consider the simple MN formed by two subsystems arranged in series with two feedback loops of regulatory signals. The MSb1 is activated by the second subsystem and the MSb2 is totally inhibited by the first subsystem when this one reaches a determinate threshold value (figure 1). The MSb1 input flux value is 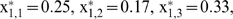 with 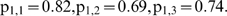 The parameter values for the integration functions of MSb2 are: 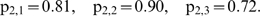 The catalytic dissipative element MSb1 is activated by the second MSb, with 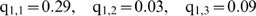 and the MSb2 is totally inhibited by MSb1, with a threshold δ = 0.18.

The initial state is
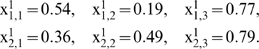
We will describe next in detail the way of obtaining the second state.

After the flux integration stage we reach an intermediary state
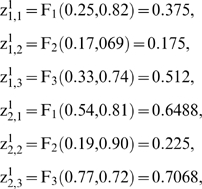
After the signal regulatory integration stage we obtain the following state
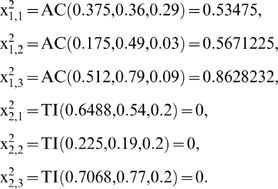
In the DMN the first metabolic subsystem will fall into a single active state, corresponding to a periodic oscillation, and the second subsystem is locked into an inactive state.
